# Measurement, dynamic evolution and influencing factors of green development efficiency in western China: Based on ecological-economic-social system

**DOI:** 10.1371/journal.pone.0290472

**Published:** 2023-12-20

**Authors:** Kun Liang, Zhongfeng Li, Li Luo

**Affiliations:** 1 School of Economics, Sichuan University, Chengdu, Sichuan, China; 2 Sichuan Academy of Social Sciences, Chengdu, Sichuan, China; 3 Sichuan Engineering Technical College, Deyang, Sichuan, China; Shanghai University of Electric Power, CHINA

## Abstract

Based on the ecological-economic-social system, green development efficiency is divided into green ecological efficiency, green economic efficiency and green social efficiency. Their corresponding indicator systems are constructed, and the Super-SBM model, Super-SBM-Undesirable model and kernel density estimation are applied to measure and analyze green development efficiency and its dynamic evolution in western China from 2007 to 2019. Tobit model is constructed and used to empirically analyze the influencing factors of the green development efficiency in western China. The study shows that: (1) green ecological efficiency and green economic efficiency in western China are generally at a low level, and mainly dragged by northwest China, while green social efficiency in western China is generally at a high level, and mainly dragged by southwest China; (2) green ecological efficiency, green economic efficiency and green social efficiency in western China all show a slight trend of first decreasing and then increasing; (3) all three sub-efficiencies of green development in western China have a decreasing trend of absolute difference, right trailing and polarization; (4) the lower green ecological efficiency in western China is due to the negative impacts from the level of government intervention, the level of economic development, and foreign direct investment. The lower green economic efficiency is due to the positive impacts from population density, the level of government intervention, the level of financial development, and foreign direct investment. The higher green social efficiency is due to the positive impacts from population density, the level of financial development, the level of economic development, and the green technological innovation. The study is based on countermeasure recommendations focusing on improving green social efficiency in southwest China, as well as green ecological efficiency and green economic efficiency in northwest China, which are of reference value to promote green development more comprehensively in western China.

## 1.Introduction

Green development efficiency is a hot topic of great interest to the academic community. Green development efficiency refers to the relationship between the amount of outputs and the amount of factor inputs for green development, reflecting the extent to which various input factors are effectively allocated and utilized in the green development process. Improving green development efficiency can enhance the capacity and quality of regional green development. At the current stage, many countries and regions in the world are facing sustainability problems such as serious ecological damage and environmental pollution, the outbreak of the energy crisis, extensive economic growth and prominent social contradictions. There is an urgent need for a green transformation of the socio-economic development mode. In 2019, the European Green Deal, officially released by the European Commission, proposed a green transformation from technology, energy, industry and transportation to the economy, with an emphasis on the protection and restoration of natural ecosystems, the sustainable use of resources and the improvement of human health [1]. In 2021, China’s Fourteenth Five-Year Plan emphasized “promoting green development and harmonious coexistence between human beings and nature” and proposed “upgrading the quality and stability of ecosystems, sustainably improving the quality of the environment, and promoting the comprehensive transformation of economic and social development”. It is evident that countries have attached importance to comprehensively promoting green development in the ecological, economic and social aspects. How to maximize the effectiveness of green development with minimal factor inputs in each of the ecosystem, economic system and social system is crucial for better and faster local green development. Existing studies can only evaluate the green development efficiency of the entire ecological-economic-social complex system, but cannot specifically reflect the green development efficiency of the ecosystem, economic system and social system, which is not conducive to the governments’ formulation of corresponding policies for these three areas. In addition, there is obvious heterogeneity among the ecosystem, economic system and social system in green operation, so it is easier to measure the green development efficiency of these three systems separately to guide practice. Western China is a key area of the national ecological security barrier and national key ecological functional area, and also contains ethnic minority areas that the central government focuses on and supports. Therefore, taking Western China as a sample can better provide realistic case support for further delineating and measuring green development efficiency based on ecological, economic and social systems. The main contributions of this study include: based on the ecological-economic-social system theory of sustainable development economics and the theoretical framework of green development, classifying green development efficiency into green ecological efficiency, green economic efficiency and green social efficiency, and constructing an indicator system for evaluating green development efficiency; measuring the bases of the green development efficiency of prefectural-level cities (and cities above) in western China, and then further analyzing their dynamic evolution characteristics and exploring their influencing factors. This study can provide some reference value for the study of green development efficiency in ecologically endowed areas, ecologically fragile areas, and areas inhabited by ethnic minorities.

## 2.Literature review

Efficiency research related to green development mainly involves green economic efficiency, green productivity, green innovation efficiency, green technological efficiency, eco-efficiency and green resource utilization efficiency [2–6]. And these efficiencies mainly belong to the efficiency of the economic system or one of its aspects, and do not completely cover all the connotations of green development efficiency. The study of green development efficiency first needs to be carried out according to the theoretical framework of green development.

From the viewpoint of the theoretical framework on which the measurement of green development efficiency is based, a considerable part of the research is based on the theoretical framework of green growth or green economy to construct a regional green development efficiency indicator system. In particular, input factors include capital, labor, energy and other factors. Desired output includes gross regional product, and non-desired output includes various types of pollutants in production. In addition, some studies have included water supply, total science and technology expenditures in the input indicators, and CO_2_ emissions in the non-expected output indicators [7–9]. In measuring urban green development efficiency, input factors include not only capital, labor and resources, but also land. Some studies have also equated green development efficiency with green total factor productivity, such as Yang and Ni (2021), Wang and Feng (2021), Yu et al. (2022), and Zhang et al. (2021) [10–13]. As the theoretical framework of green development becomes more and more mature, some studies have been based on the ecological-economic-social composite system, considering the input-output relationship in the green development of the whole composite system. According to the production function in economics, scholars chose capital, labor, technology, resources as input indicators, and set the desired outputs as economic gains. Subsequent studies have added social gains, ecological gains or technological gains to the desired outputs [14]. Most of the non-desired outputs are environmental pollutants and CO_2_. Some individual studies have also added indicators of negative social outputs. However, in this kind of indicator system, the input indicators are selected from the production factors of the economic system, and the output indicators are selected from the three types of output results of the economic system, ecosystem and social system. Therefore, this kind of indicator system is obviously flawed in that the inputs and outputs do not form a correspondence with each other.

In terms of the structure of the indicator system, there is a consensus among existing studies that the construction of the indicator system for green development efficiency mainly uses the structure of “inputs―desired outputs―non-desired outputs”. In particular, the non-desired outputs mainly take into account carbon neutral concerns about carbon emissions, environmental pollution in production and life, and so on [15–18].

With the deepening of green development research, green development explicitly emphasizes the three dimensions of ecological, economic and social sustainability [19]. Although the academic community has not yet paid attention to the structure of green development efficiency based on the framework of ecological-economic-social systems to delineate the efficiency of green development, for the study of green development efficiency, many scholars distinguished the efficiency of the economic system, social system and ecosystem to measure.

Firstly, about the measurement of green economy efficiency. This efficiency takes into account not only the traditional “good” outputs of economic activity, but also the “bad” outputs that have a negative environmental impact. Existing studies mainly use capital, labor and energy as input factors, and the indicators of non-desired outputs usually choose emissions of waste gas, wastewater, solid waste and CO_2_. The input indicators in some studies also include technology, land, and water [20–23].

Secondly, about the measurement of eco-efficiency. The object of measuring this efficiency is economic system and ecosystem. The content of measuring is the relationship between the value return of the economic system as well as the resource and environmental cost paid by the ecosystem [24,25]. Thus, the essence of eco-efficiency is ecological-economic efficiency [26]. The common measurement methods can be summarized as a value-impact ratio and an efficiency model. These efficiency indicators mainly took capital, human resources, natural resources and environmental indicators as inputs and environmental impacts as outputs [27,28]. Liu (2022) constructed the ecological governance efficiency indicators of the Yellow River basin with water resource use, labor, ecological governance capital, and government attention as governance inputs, economic development and ecological governance effectiveness as desired outputs, and ecological water pollution as non-desired outputs [29]. Sun (2022) measured the ecological capital efficiency of the Yellow River basin with ecological resource stock and ecological environment quality as input indicators as well as ecological service value and ecological environment condition as output indicators [30].

Thirdly, on the concept and measurement of social efficiency. Social efficiency is the social system to obtain the maximum social output with the minimum input [31]. According to Commons’ definition of efficiency, social efficiency is the ratio of social benefits to social costs [32]. Social efficiency consists of two levels, namely “efficiency of social costs and benefits” from the administrative perspective, and “efficiency of social resource allocation” from the economic perspective [33]. Existing studies have addressed social efficiency in renewable energy policy, air services, and traffic management [34–36]. Luo et al. (2021) constructed the indicator system of social governance efficiency by taking pollution control investment, social petitions and environmental regulations as inputs, industrial SO_2_ and wastewater treatment rate and accepted social petitions as expected outputs, and environmental administrative penalty cases, industrial SO_2_ and wastewater emissions after treatment as non-desired outputs [37].

In terms of green development efficiency measurement methods, measurement models have made great progress and are more mature, with SBM model, Super-SBM model, Super-SBM-Undesirable model as the main ones. Some researches in the field have also used Window DEA model, three-phase Window DEA model, Super-efficiency EBM model, and non-directional EBM-Undesirable and so on [22,23,38–41].

There are many factors affecting green development efficiency, and the ones that have attracted more attention from academics are the level of financial development or green finance, foreign direct investment, the level of economic development, the structure of industry, technological innovation or green technological innovation, natural resources, and the degree of government intervention and so on [42–46]. However, the above factors may positively or negatively affect green development efficiency.

Green development is essentially the green development of economic system, social system and ecosystem. In the early research, there was a confusion in the indicator system between green development efficiency and green economic efficiency. Only in recent years have academics begun to pay attention to the efficiency of ecosystems and social systems in green development. The relevant research is only at the preliminary stage. From the green development efficiency indicator systems constructed by existing studies, the measurement objects are still mainly economic systems, and minority studies only include social and ecological indicators in the output parts. The measurement objects of green development efficiency in existing studies do not all include ecosystem, economic system and social system. At the same time, if only green development efficiency is calculated for the ecological-economic-social complex system, the resulting efficiency is calculated by sharing a common production frontier within the complex system. This conceals the great differences in production conditions and technologies of each of the ecosystem, economic system, and social system in the green development process. Future research is necessary to delineate the structure of green development efficiency in terms of ecological, economic and social subsystems, and to measure green economic efficiency, green ecological efficiency, and green social efficiency, respectively.

## 3.Research design

### 3.1. Theoretical framework

The object of study in the sustainability economics is the ecological-economic-social complex system [47]. According to system theory, there are obvious differences between different systems in terms of elements, structures and functions. Because of the integrity of the system itself and the differences between systems, the means, methods and paths to promote green development in the ecological, social and economic fields vary from region to region. There are obvious differences in the green development capacity and green development performance in the ecological, economic and social aspects. The “three layers” theoretical framework of green development is based on the ecosystem, economic system and social system [48]. In practice, many countries are fully promoting green development in the three major areas of ecology, economy and society. Then, the ecosystem, economic system and social system can be used as the theoretical and practical basis for the division of green development efficiency components in this study.

**Fig 1:**
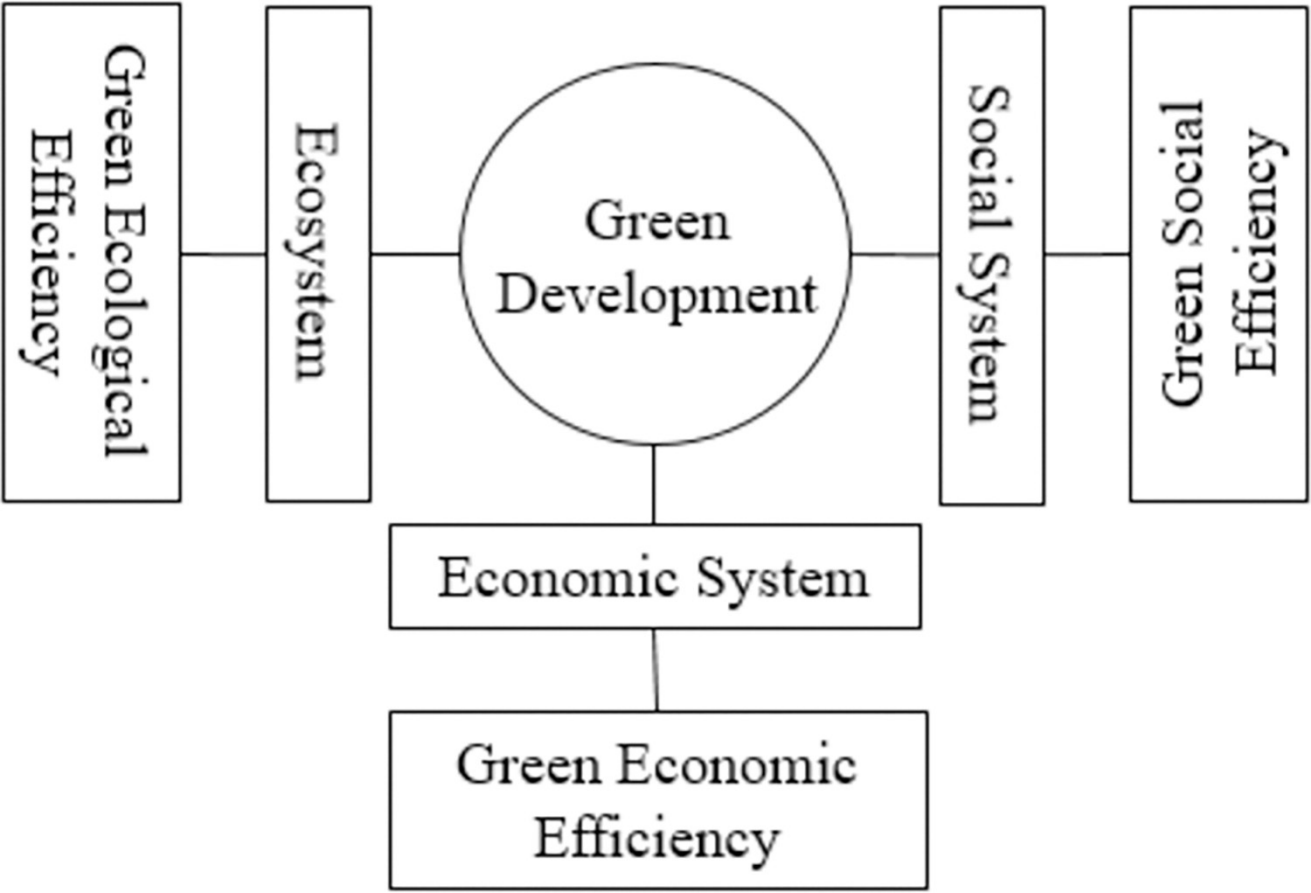
The theoretical framework of green development efficiency.

Green development efficiency reflects the input-output relationship of the ecological-economic-social system in the process of green development. The three subsystems of economy, ecology and society show differences in the input elements and their degree of utilization in green development due to the differences in production method or operation method. Moreover, the outputs of the three systems are green economic outcomes, ecological welfare and wealth, and social well-being, so the green development efficiency can be further analyzed by subdividing them into green economic efficiency, green ecological efficiency, and green social efficiency (Fig 1). (1) From the connotation of the three kinds of efficiency, green ecological efficiency is the relationship between factor inputs in the ecosystem and its supply of ecological well-being, indicating the reproductive capacity of ecosystem services in the process of ecological protection. Green economic efficiency considers the use of energy resources in economic activities and the environmental costs they generate, and is the input-output relationship of green economic development, indicating the degree of green utilization of production factors and the development capacity of green economic. Green social efficiency is the input-output relationship of factors in the process of green social service and governance, which indicates the capacity of green social service and governance. These three efficiencies represent the green development capacity of the region in ecological, economic and social aspects respectively. In particular, ecosystem services are the benefits that ecosystems provide to humans with their own functions, and therefore are regarded as the outputs of ecosystems by ecological economics scholars. The Human Development Index proposed by the United Nations Development Program in the *Human Development Report 1990* mainly took life expectancy, education level, and quality of life as the base variables. *The 2030 Agenda for Sustainable Development* has proposed social sustainable development goals in terms of poverty, health, education, gender equality, and environmental health [49,50]. These provide a reference for the selection of input and output indicators for narrowly defined social system. (2) In terms of the relationship between the three types of efficiency, each of the three systems corresponding to green ecological efficiency, green economic efficiency and green social efficiency has different production frontiers in itself. In addition, the three systems themselves are juxtaposed rather than subordinated. Then, green ecological efficiency, green economic efficiency, and green social efficiency are also three kinds of efficiencies in parallel. (3) From the perspective of the nature of efficiency, achieving green development efficiency is the green development system to obtain the maximum output under certain input conditions or to achieve the minimum input under certain output, which in essence belongs to Pareto efficiency. Then, green ecological efficiency, green economic efficiency, and green social efficiency, as sub-efficiencies of green development efficiency, also belong to the category of Pareto efficiency.

### 3.2. Indicator system design

In accordance with the theoretical framework of green development efficiency and the variable setting of the production function in economics, by drawing on relevant research results at home and abroad, based on data availability and practicality, the indicator system about green ecological efficiency, green economic efficiency and green social efficiency is designed. The following three points need to be clarified: (1) Green ecological efficiency uses ecosystem services value as desired output, in which the two service values of resource provision and cultural recreation are involved in the production activities of the economic system. The value quantity of these two services is included in the accounting of the output of the economic system. In this paper, the final outputs of regulation services and support services that provide human well-being are used as the content of ecosystem output value accounting. In this paper, the 2015 equivalent factor table obtained from Xie et al. (2015) is used based on the Chinese reality, and the practice of Cheng et al. (2019) and Liu et al. (2020) is referred to calculate ecosystem service value [51–53]. (2) Existing studies have not reached a consensus on whether ecological protection capital investment is included in the input indicators of green ecological efficiency. Only provincial-level data on ecological construction and protection capital investment are available. Data on ecological protection capital investment at the prefecture-level municipalities are not available. The relevant yearbooks have not yet counted provincial-level data on ecological construction and protection investment between 2007 and 2010. In view of this, this paper does not include ecological protection capital indicators. (3) Considering that China’s National Poverty Eradication Conference in February 2021 announced that China has eliminated absolute poverty, and gender equality lacks data support, the green social efficiency described in this paper mainly involves health care, education, and environmental health, which correspond to the three basic components of the Human Development Index. Among the green social efficiency indicators, social service and governance funds mainly include the completed investment in fixed assets for urban municipal utility construction and financial expenditures on education. Social service and governance personnel mainly include residential services and other services workers, education workers, health and social workers, as well as public administration, social security and social organization personnel. The compositions of indicators of green ecological efficiency, green economic efficiency and green social efficiency in western China are s shown in Table 1.

**Table 1:** Compositions of green development efficiency indicators in western China.

Efficiency categories	Indicators	Indicator categories	Compositions
Green ecological efficiency	Input indicators	Ecological capital investment	Total ecological land (hm^2^)
		Ecological protection labor	Number of employees in the water, environment and public facilities management industry (10^4^ people)
	Output indicator	Ecological output	Ecosystem service value (10^4^ RMB)
Green economic efficiency	Input indicators	Capital input	Capital stock (10^4^ RMB)
		Labor input	Number of employees in units at the end of the year (10^4^ people)
		Energy input	Electricity consumption of the whole society (10^4^ kWh)
	Desired output indicator	Economic output	Real GDP (10^4^ RMB)
	Non-desired output indicators	Pollution discharge	CO_2_ emissions(t), SO_2_ emissions(t), COD emissions(t)
Green social efficiency	Input indicators	Capital input	Social services and governance funds (10^4^ RMB)
		Labor input	Number of social service and governance staff (10^4^ people)
		Pollutants in daily life	Sewage discharge volume (10^4^ m^3^), Domestic waste removal volume (10^4^ t)
	Output indicator	Social service & governance output	Number of doctors per 10^4^ people (persons), Ratio of elementary school teachers to students (per 10^4^ people), Greening coverage of built-up areas (hm^2^), Total volume of sewage treatment (10^4^ m^3^), Volume of domestic waste disposal (10^4^ t)

### 3.3. Super-SBM model and Super-SBM-Undesirable model

In this paper, Super-SBM model is applied to measure green ecological efficiency and green social efficiency, and Super-SBM-Undesirable model to measure green economic efficiency. The super efficiency model uses a linear programming approach to estimate efficiency value by measuring the weights of the input-output ratio, hence the name ray efficiency. Tone (2001) first proposed an efficiency estimation model based on difference variables, which is called SBM model by considering the slacks between input and output terms in a non-ray estimation, while the estimated efficiency value is between 0 and 1 [54]. However, under this model, it still produces the problem that the SBM efficiency values of multiple decision making units (DMU) are the same as 1, so Tone (2002) proposes a modified slack-based measure of super-efficiency model [55]. Super-SBM model is based on SBM model to estimate the super efficiency value of DMU, which can solve the problem that the SBM efficiency values of multiple DMUs are the same as 1. At the same time, since the Super-SBM model is to estimate the efficiency value in a non-ray way, it does not create the problem that cannot be estimated like MDEA. Suppose there are j (j = 1,…, n) DMUs, each DMU has inputs and outputs, using i (i = 1,…, m) kinds of inputs *x*_*i*_ to get r (r = 1,…, s) kinds of outputs *y*_*r*_. The Super-SBM model is as follows:

$$min\;\delta =\frac{\frac{1}{m}\sum_{i=1}^{m}\frac{{\bar{x}}_{i}}{x_{i0}}}{\frac{1}{s}\sum_{r=1}^{s}\frac{{\bar{y}}_{r}}{y_{r0}}}$$

$$s.t.\left\{ \begin{array}{c} \bar{x}\geq \sum_{j=1,\neq 0}^{n}\lambda_{j}x_{j}\\ \bar{y}\geq \sum_{j=1,\neq 0}^{n}\lambda_{j}y_{j}\\ \bar{x}\geq x_{0},\bar{y}\leq y_{0}\\ \bar{y}\geq 0,\lambda \geq 0 \end{array}\right.$$ (1)

In Eq (1), *λ*_*j*_ denotes the weights of the DMU. Adding the Super-SBM model to the concept of variable scale payoff, the model is as follows:

$$min\;\delta =\frac{\frac{1}{m}\sum_{i=1}^{m}\frac{{\bar{x}}_{i}}{x_{i0}}}{\frac{1}{s}\sum_{r=1}^{s}\frac{{\bar{y}}_{r}}{y_{r0}}}$$

$$s.t.\left\{ \begin{array}{c} \bar{x}\geq \sum_{j=1}^{n}\lambda_{j}x_{j}\\ \bar{y}\leq \sum_{j=1}^{n}\lambda_{j}y_{j}\\ \sum_{j=1}^{n}\lambda_{j}=1\\ \bar{x}\geq x_{0},\bar{y}\leq y_{0}\\ \bar{y}\geq 0,\lambda \geq 0 \end{array}\right.$$ (2)

After Tone proposed the Super-SBM model, scholars improved it into the Super-SBM-Undesirable model. There are obvious advantages of the Super-SBM-Undesirable model: first, the model can solve the problem that multiple DMUs with efficiency value of 1 in the SBM model need to be ranked; second, relative to the radial DEA model, the model breaks the two assumptions of the same (equal) proportional substitution of input factors and the same direction of undesired output and desired output; third, the model takes into account the case of undesired output relative to the Super-SBM model. It is those advantages of the model that make the measured green economic efficiency more accurate. Suppose there are j (j = 1,…, n) DMUs, each with inputs, desired outputs and undesired outputs, using i (i = 1,…, m) kinds of inputs *x*_*i*_ to obtain r (r = 1,…, s_1_) kinds of desired outputs $y_{r}^{g}$ and k (k = 1,…, s_2_) kinds of undesired outputs $y_{k}^{b}$. The production possibility set P (or environmental technology) is:

$$\bar{P}=\{\left(\bar{x},{\bar{y}}^{g},{\bar{y}}^{b}\right)|\begin{array}{c} \bar{x}\geq \sum_{j=1,\neq 0}^{n}\lambda_{j}x_{j}\\ {\bar{y}}^{g}\leq \sum_{j=1,\neq 0}^{n}\lambda_{j}y^{g}\\ {\bar{y}}^{b}\geq \sum_{j=1,\neq 0}^{n}\lambda_{j}y^{b}\\ {\bar{y}}^{b}\geq 0,\lambda \geq 0 \end{array}\}$$ (3)

In Eq (3), *λ*_*j*_ denotes the weights of DMU. Based on the production possibility set, the Super-SBM-Undesirable model is as follows:

$$\rho^{*}=min\;\rho =\left[\frac{1}{m}\sum_{i=1}^{m}\frac{{\bar{x}}_{i}}{x_{i0}}\right]/\left[\frac{1}{s_{1}+s_{2}}(\sum_{r=1}^{s_{1}}\frac{{\bar{y}}_{r}^{g}}{y_{r0}^{g}}+\sum_{k=1}^{s_{2}}\frac{{\bar{y}}_{k}^{b}}{y_{k0}^{b}})\right]$$

$$s.t.\left\{ \begin{array}{c} \bar{x}\geq \sum_{j=1,\leq \neq 0}^{n}{\lambda_{j}x}_{j}\\ {\bar{y}}^{g}\leq \sum_{j=1,\leq \neq 0}^{n}\lambda_{j}y_{j}^{g}\\ {\bar{y}}^{b}\leq \sum_{j=1,\leq \neq 0}^{n}\lambda_{j}y_{j}^{b}\\ \bar{x}\geq x_{0},{\bar{y}}^{g}\leq y_{0}^{g},{\bar{y}}^{b}\geq y_{0}^{b}\\ \lambda >0 \end{array}\right.$$ (4)

In Eq (4), the value of the objective function ρ* may be >1 or ≤1, and the larger ρ* indicates the more efficient the DMU. In the specific software operation, the Super-SBM model used in this paper selects “variable payoffs to scale” and “global reference”. The principle of the global reference is to use the sum of all periods as the reference set, with each period referring to the same frontier. The efficiency value calculated using the global reference is the global efficiency value, not the current period efficiency value. Thus, the global efficiency value facilitates longitudinal time comparisons between the efficiency of each region in each year.

### 3.4. Kernel density estimation

Kernel density estimation is an important nonparametric estimation method that can describe the distribution characteristics of green development efficiency of prefecture-level cities in western China with a continuous density curve. The dynamic evolution of the distribution characteristics of green development efficiency in the region can be identified by longitudinally comparing the kernel density curves of samples from multiple periods in the same region. Specifically, the kernel density curves of green development efficiency are generated by the following functions:

$$f(y)=\frac{1}{Nh}\sum_{i=1}^{N}K(\frac{y_{i}-y}{h})$$ (5)

$$K(x)=\frac{1}{\sqrt{2\pi }}exp(-\frac{x^{2}}{2})$$ (6)

Where *K*(*x*) represents the kernel density function, which describes the weights occupied by all sample points *y*_*i*_ in the y neighborhood, N represents the number of observations, and h represents the window width of the kernel density estimation. In terms of kernel density function selection, the commonly used kernel density functions are Gaussian kernel, Epanechnikov kernel, double-angle kernel, triangular kernel, etc. However, in general, the selection of different kernel density functions has little effect on the estimation results, so this paper is based on the Epanechnikov kernel function to calculate.

### 3.5. Data sources

The data of prefecture-level cities in western China were obtained from the China City Statistical Yearbook and China Urban-Rural Construction Statistical Yearbook from 2008 to 2020 [56,57]. The planted area, unit area yield, and total production of the three major grain crops of wheat, corn, and soybeans in each province of western China were obtained from the provincial statistical bureaus. The average grain prices were obtained from the National Compilation of Agricultural Cost-benefit Information (2016) [58]. In this paper, the 30m land use classification products published in Earth System Science Data by Yang and Huang of Wuhan University were used [59]. The data of urban CO_2_ emissions in western China were obtained from the Institute of Public and Environmental Affairs (IPE) database [60]. Considering that the 17th Congress of the Communist Party of China in 2007 made the construction of ecological civilization as one of the goals to realize the construction of a moderately prosperous society, and there was a serious lack of data for some indicators after 2019, the year interval of the samples in this paper was selected as 2007–2019.

Considering the availability of data, cities with serious missing data, such as Tibet Autonomous Region, Bijie City and Tongren City, and newly established cities in recent years, such as Hami City, are not included in the samples in this paper. In the samples of this paper, the nearest neighbor interpolation method is used to make up for the missing data in the middle years, and the data in the neighboring years are used to make up for the small amount of missing data in the first and last years of the observation period. Through matching, s balanced panel data for a total of 1079 samples of 84 prefecture-level cities (including municipality) in western China from 2007 to 2019 are obtained in this paper (Table 2).

**Table 2:** 84 cities in western China.

Southwest China	Northwest China
(1) Guangxi Zhuang Autonomous Region: Nanning, Liuzhou, Guilin, Wuzhou, Beihai, Fangchenggang, Qinzhou, Guigang, Yulin, Baise, Hezhou, Hechi, Laibin, Chongzuo; (2) Chongqing Municipality; (3) Sichuan Province: Chengdu, Zigong, Panzhihua, Luzhou, Deyang, Mianyang, Guangyuan, Suining, Neijiang, Leshan, Nanchong, Meishan, Yibin, Guang’an, Dazhou, Ya’an, Bazhong, Ziyang; (4) Guizhou Province: Guiyang, Liupanshui, Zunyi, Anshun; (5) Yunnan Province: Kunming, Qujing, Yuxi, Baoshan, Zhaotong, Lijiang, Pu’er, Lincang.	(1) Inner Mongolia Autonomous Region: Hohhot, Baotou, Wuhai, Chifeng, Tongliao, Erdos, Hulunbeier, Bayannur, Ulanqab; (2) Shaanxi Province: Xi’an, Tongchuan, Baoji, Xianyang, Weinan, Yan’an, Hanzhong, Yulin, Ankang, Shangluo; (3) Gansu Province: Lanzhou, Jiayuguan, Jinchang, Baiyin, Tianshui, Wuwei, Zhangye, Pingliang, Jiuquan, Qingyang, Dingxi, Longnan; (4) Qinghai Province: Xining; (5) Ningxia Hui Autonomous Region: Yinchuan, Shizuishan, Wuzhong, Guyuan, Zhongwei; (6) Xinjiang Uygur Autonomous Region: Urumqi, Karamay.

## 4. Measurements of green development efficiency in western China

### 4.1. Characteristics of green ecological efficiency in western China

In this paper, the green ecological efficiency of 84 cities in western China from 2007 to 2019 is measured using the Super-SBM model of Eqs (1) to (2). Table 3 shows the interval distribution characteristics of green ecological efficiency for each sample during the observation period. Specifically:

First, from the interval distribution of samples, there are 6, 47, 21 and 10 samples in four levels from low level to high level in 2007, indicating that 55.95% of the samples in western China are in the lower level of green ecological efficiency in 2007. In 2019, the green ecological efficiency samples at the lower level accounted for 48.81% in western China. The number of samples with green ecological efficiency over 0.50 increased by only 3 compared to 2007, indicating that the overall level of green ecological efficiency in western China is not high, and the change within each interval is small, with only a slight increase.

Second, from the perspective of north and -south geographies, the last two columns of Table 3 show the overall mean value and sample interval distribution of green ecological efficiency in southwest China and northwest China in 2019. In particular, the green ecological efficiency of the samples in the southwest China is mainly concentrated in the lower and higher levels, and the samples with green ecological efficiency over 0.50 account for 57.78% in southwest China, while 56.41% of the samples in northwest China are mainly concentrated in the lower level interval, which shows that the green ecological efficiency of the samples in southwest China is generally better than that in northwest China.

Third, in terms of the geographical distribution of high and low values, in 2007, 6 prefecture-level cities in northwest China were at a low level, while 8 prefecture-level cities in southwest China and 2 prefecture-level cities in northwest China were at a high level. In 2019, 9 prefecture-level cities in northwest China were at a low level, while 3 prefecture-level cities in northwest China and 8 prefecture-level cities in southwest China were at a high level. It can be seen that the areas with the low level of green ecological efficiency is distributed in northwest China, and the areas with its high level is mainly distributed in southwest China.

**Table 3:** Distribution intervals of green ecological efficiency in western China.

Level	Index range	2007	2013	2019		
				Overall	Southwest China	Northwest China
High level	(0.75,2)	10	7	11	8	3
Medium high level	(0.50,0.75]	21	21	23	18	5
Medium low level	(0.25,0.50]	47	44	41	19	22
Low level	[0,0.25]	6	12	9	0	9
Mean value		0.4802	0.4367	0.4855	0.5630	0.3962

### 4.2. Characteristics of green economic efficiency in western China

In this paper, the green economic efficiency of 84 cities in western China from 2007 to 2019 is measured using the Super-SBM-Undesirable model with Eqs (3) to (4). Table 4 shows the interval distribution characteristics of green economic efficiency for each sample during the observation period. Specifically:

**Table 4:** Distribution intervals of green economic efficiency in western China.

Level	Index range	2007	2013	2019		
				Overall	Southwest China	Northwest China
High level	(0.75,2)	14	6	19	14	5
Medium high level	(0.50,0.75]	9	7	12	8	4
Medium low level	(0.25,0.50]	52	54	40	19	21
Low level	[0,0.25]	9	17	13	4	9
Mean value		0.4972	0.3917	0.5396	0.6261	0.4399

First, from the interval distribution of the samples, there are 9, 52, 9 and 14 samples in four levels from low level to high level in 2007, indicating that 61.90% of the samples in western China are in the lower level of green economic efficiency in 2007; the samples in the lower level of green economic efficiency in 2019 account for 47.62% in western China, and the green economic efficiency over 0.50 sample increased by 8 compared to 2007, indicating that the overall green economic efficiency in western China is at the lower level, with a slight increase in the interval.

Second, from the perspective of north and south geographies, the last two columns of Table 4 show the overall mean value and sample interval distribution of green economic efficiency in southwest China and northwest China in 2019. In particular, the overall mean value of green economic efficiency in southwest China is 0.6261, which is higher than that e of 0.4399 in northwest China. The green economic efficiency of the samples in southwest China is mainly concentrated in the lower level and high level. The samples with green economic efficiency over 0.50 account for 48.89% in southwest China, while 53.85% of the samples in northwest China are mainly concentrated in the lower level, which shows that green economic efficiency of the samples in southwest China is generally better than that in northwest China.

Third, in terms of the geographical distribution of high and low values, in 2007, 3 prefecture-level cities in southwest China and 6 prefecture-level cities in northwest China were at a low level. 8 prefecture-level cities in southwest China and 6 prefecture-level cities in northwest China were at a high level. In 2019, 4 prefecture-level cities in southwest China and 9 prefecture-level cities in northwest China were at a low level. 5 prefecture-level cities in northwest China were at a high level. Other provinces in southwest China except Chongqing have prefecture-level cities at the high level. It can be seen that the areas with the low level of green economic efficiency are mainly distributed in northwest China, and the areas with its high level are mainly distributed in southwest China.

### 4.3. Characteristics of green social efficiency in western China

In this paper, the Super-SBM model using Eqs (1) to (2) is used to measure the green social efficiency of 84 cities in western China from 2007 to 2019. Table 5 shows the interval distribution characteristics of green social efficiency for each sample during the observation period. Specifically:

**Table 5:** Distribution intervals of green social efficiency in western China.

Level	Index range	2007	2013	2019		
				Overall	Southwest China	Northwest China
High level	(0.75,2)	30	16	25	12	13
Medium high level	(0.50,0.75]	14	20	26	11	15
Medium low level	(0.25,0.50]	26	46	33	22	11
Low level	[0,0.25]	14	2	0	0	0
Mean value		0.5952	0.5374	0.6361	0.6781	0.5997

First, from the interval distribution of samples, there were 14, 26, 14 and 30 samples in four levels from low level to high level in 2007, indicating that 35.71% of the samples in western China were at high level of green social efficiency and 30.95% were at lower level in 2007. In 2019, all samples in western China have been at above lower level. Green social efficiency over 0.50 accounted for 60.71% of the samples in western China, an increase of 7 compared to 2007, indicating that the green social efficiency in western China is generally at the higher level, with some increase in the interval.

Second, from the perspective of north and south geographies, the green social efficiency of the samples in southwest China is mainly concentrated in the lower level. The samples with green social efficiency over 0.50 account for 51.11% in southwest China, while the samples in northwest China are relatively evenly distributed among the lower, higher and high levels, which shows that the green social efficiency of the samples in northwest China is generally better than that in southwest China.

Third, in terms of the geographical distribution of high and low values, in 2007, 12 prefecture-level cities in southwest China and 2 prefecture-level cities in northwest China are at a low level, while 11 prefecture-level cities in southwest China and 19 prefecture-level cities in northwest China were at the high level. In 2019, no prefecture-level cities in southwest China and northwest China were at the low level. The similar number of prefecture-level cities is at the high level. It can be seen that the areas with the low level of green social efficiency at the beginning of the observation period are mainly in southwest China, while the areas with the high level are mainly distributed in northwest China. The areas with the high level of green social efficiency at the end of the observation period are distributed in similar numbers in southwest China and northwest China.

## 5. Dynamic evolution of green development efficiency in western China

In order to more intuitively demonstrate the changing trends of green ecological efficiency, green economic efficiency, and green social efficiency in each region of western China, the corresponding time-series trend maps are drawn in this paper. In order to describe the dynamic changes of the three efficiencies in more details, in this paper, 2007, 2013 and 2019 are selected to draw kernel density maps based on the calculation of Eq (5) to explore the dynamic characteristics of the distribution of the three efficiencies in four aspects: location, dynamics, extensibility and polarization trend of each city in western China. In the kernel density diagram, the X-axis indicates the value of green development sub-efficiency of each city in western China, and the Y-axis indicates the relative frequency of this efficiency value.

### 5.1. Dynamic evolution of green ecological efficiency in western China

**Fig 2:**
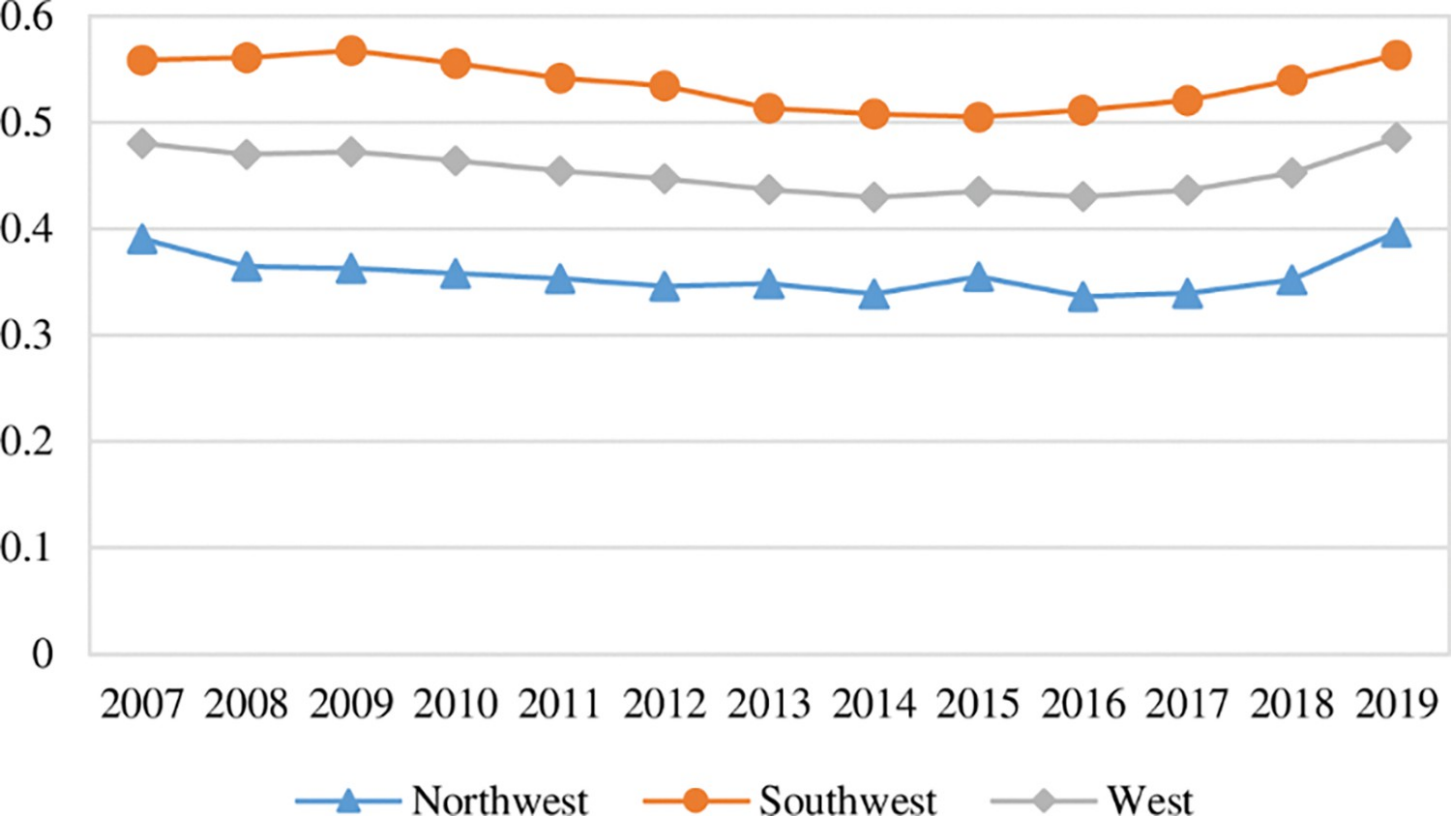
Trend of green ecological efficiency in western China.

Fig 2 illustrates the time-series trends of green ecological efficiency in each region of western China. It can be seen that:

First, from the overall mean value change, the mean value of green ecological efficiency in western China was 0.4802 in 2007, which declined to 0.4294 in 2014, and rose to 0.4855 in 2019, indicating that the mean value of green ecological efficiency in western China showed a slight fluctuation trend and an evolutionary process of first decreasing and then increasing. The same trend of change also exists in southwest China and northwest China. The ecological functioning of the ecosystem will not be significantly accelerated or slowed down in the short term. The performance of the input-output relationship reflected by green ecological efficiency will not have large fluctuations, but will be relatively stable or slightly fluctuating.

Second, in terms of regional distribution, during the observation period, the mean value of green ecological efficiency in southwest China was in the range of (0.5050, 0.5675). The mean value of green ecological efficiency in northwest China was in the range of (0.3361, 0.3962). The mean value of green ecological efficiency in western China was in the range of (0.4294, 0.4855). The green ecological efficiency of southwest China is higher than the average level of western China. The gap with western China remains relatively stable. The green ecological efficiency in northwest China is lower than the average of western China. The gap with western China remains relatively stable. In 2019, the overall mean value of green ecological efficiency in southwest China is 0.5630, which is higher than the overall mean value of 0.3962 in northwest China. Compared with northwest China, southwest China has more superior natural geographical conditions and ecological endowment conditions. The ecosystem services such as forests, wetlands, and water systems have higher output values. These types of ecological land account for a higher proportion of the local land area, while the area of bare land with low ecosystem service value is less, so the green ecological efficiency of southwest China is relatively higher than that of northwest China.

**Fig 3:**
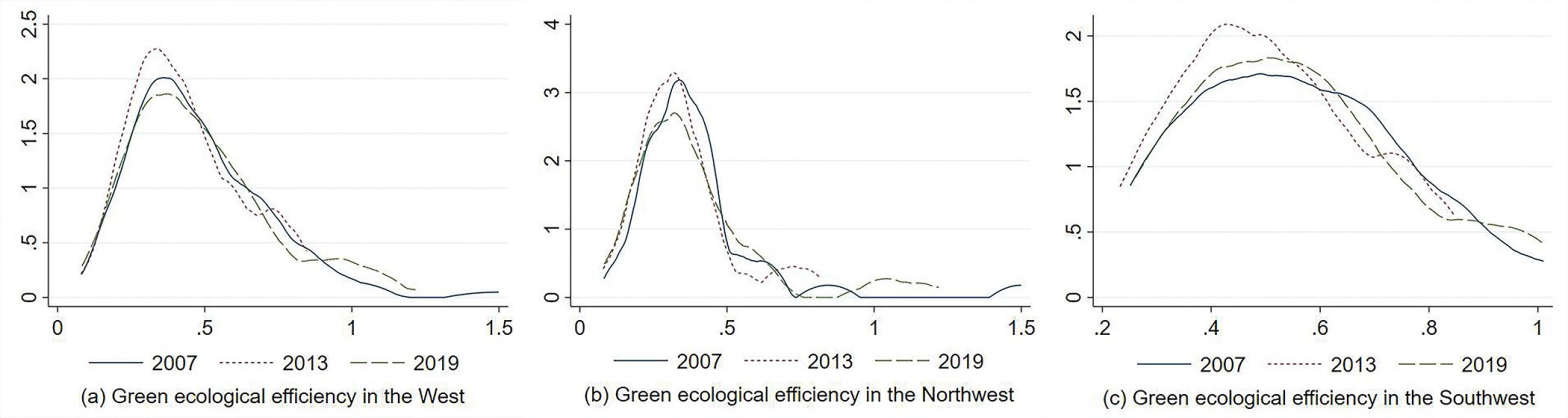
Kernel density curve of green ecological efficiency in western China.

Fig 3 shows the kernel density curves of green ecological efficiency in different regions of western China in different years. The specific distribution dynamics are characterized as follows:

First, the center of the distribution curve in western China did not shift significantly during the observation period. The height of its main peak mainly showed an evolutionary process of first rising and then falling. The width of its curve showed an evolutionary process of first narrowing significantly and then widening slightly, with a slight increase in the distribution of the right side of the curve. This indicates a slight increase in the overall trend of green ecological efficiency in western China and a trend of slightly narrowing of their absolute differences.

Second, the distribution curve of green ecological efficiency in western China has an obvious “right trailing” phenomenon. Its distribution extension shows a certain degree of contraction, which means that the gap between cities with high green ecological efficiency and the mean of cities in western China has been reduced.

Third, in terms of the evolution process of wave peaks, the green ecological efficiency distribution in western China has undergone the evolution process of “single peak—double peaks—single peak”, although the polarization characteristics tend to weaken. In 2007, there was mainly one main single peak in the distribution of green ecological efficiency in western China. Then there was a lower side peak on the right side of the curve in addition to one main peak in 2013. Then there was no obvious side peak on the right side of the curve in 2019. The polarization was not obvious, which indicates that there was a slight gradient change in the green ecological efficiency in western China during the observation period. It is generally free of extremes.

From Fig 3B and 3C, it can be seen that the centers of the distribution curves in both northwest China and southwest China are not significantly displaced. The main peaks of the curves in northwest China show a decreasing trend, while the main peaks of the curves in southwest China show a rising and then decreasing trend. The absolute differences of green ecological efficiency in northwest China show a slightly narrowing trend, while there is no change in southwest China. The right trailing phenomenon exists in northwest China while the right trailing phenomenon in southwest China is not obvious. There is no obvious polarization of green ecological efficiency in both regions.

### 5.2. Dynamic evolution of green economic efficiency in western China

**Fig 4:**
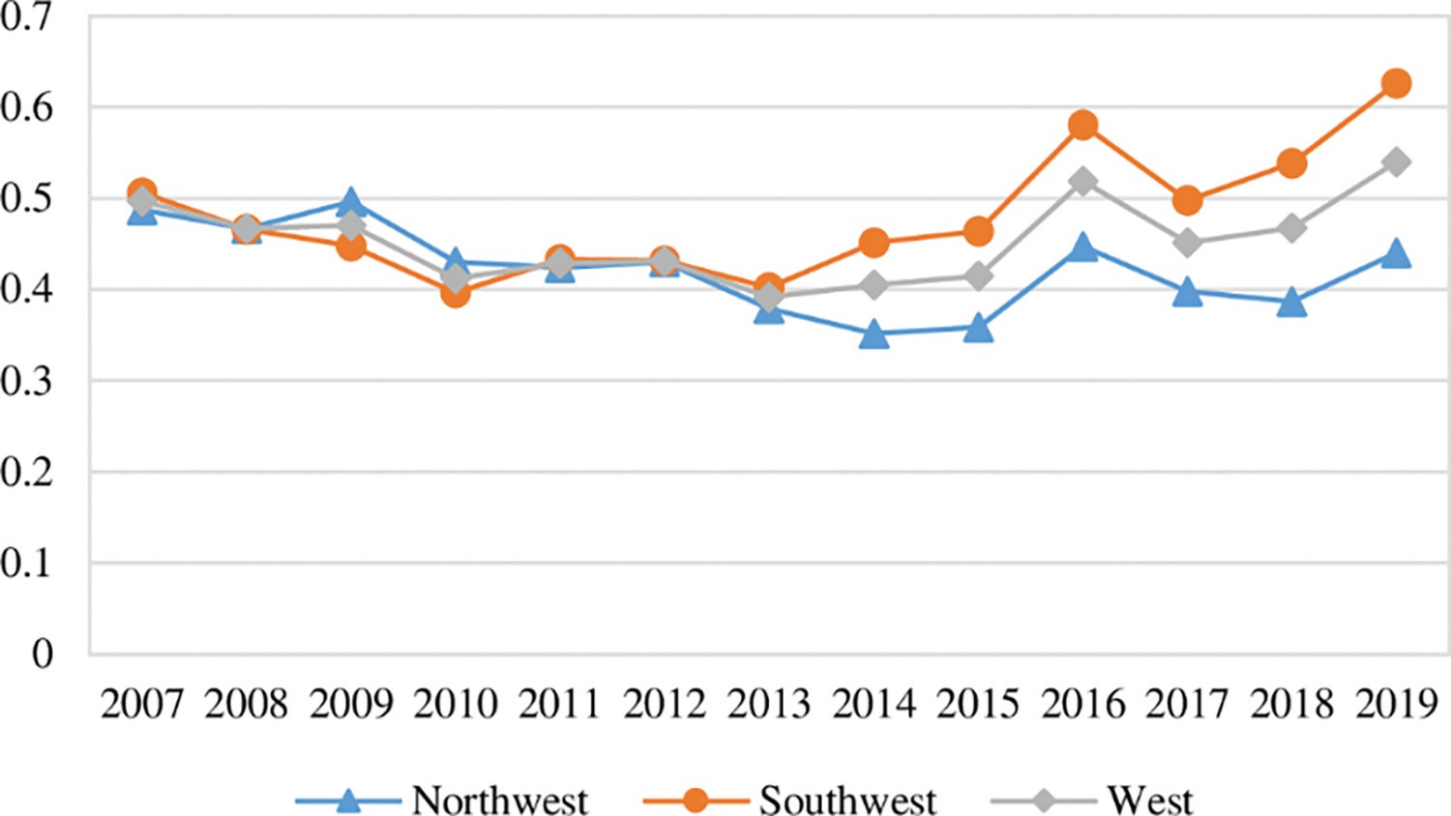
Trend of green economic efficiency in western China.

Fig 4 illustrates the time-series trend of green economic efficiency in each region of western China. It can be seen that:

First, in terms of the overall mean value change, the mean of green economic efficiency in western China was 0.4972 in 2007, which declined to 0.3917 in 2013, and increased to 0.5396 in 2019, indicating that the mean of green economic efficiency in western China showed a fluctuating trend and an evolutionary process of first decreasing and then increasing. The same trend of change existed in southwest China and northwest China. However, in the later part of the observation period, green economic efficiency in southwest China increased rapidly and was higher than its mean in 2007, while green economic efficiency in northwest China increased more slowly and was lower than its mean in 2007.

Second, in terms of regional distribution, during the observation period, the mean of green economic efficiency in southwest China is in the range of (0.4025, 0.6261). The mean of green economic efficiency in northwest China is in the range of (0.3516, 0.4872). The mean of green economic efficiency in western China is in the range of (0.3917, 0.5396). The green economic efficiency of southwest China is higher than the mean of western China. The green economic efficiency of northwest China is lower than the mean of western China. The gap between the green economic efficiency of southwest China and northwest China was smaller before 2012. The gap between the green economic efficiency of southwest China and northwest China continued to expand after 2012. In 2019, the overall mean of the green economic efficiency of southwest China was 0.5630, which was higher than the overall mean of 0.3962 in northwest China.

**Fig 5:**
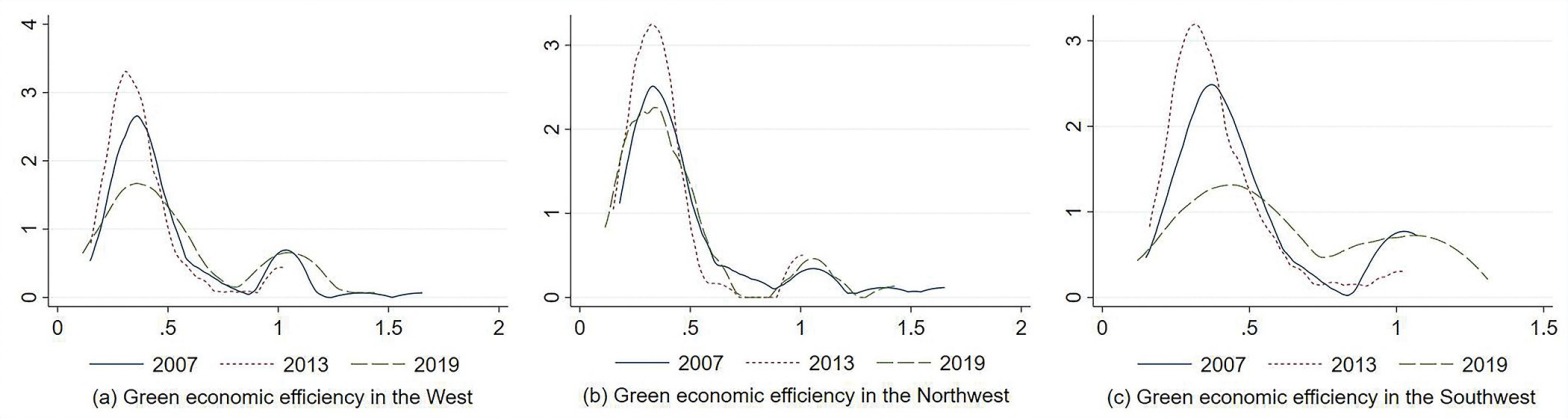
Kernel density curve of green economic efficiency in western China.

Fig 5 shows the kernel density curves of green economic efficiency in different regions of western China in different years. The specific distribution dynamics are characterized as follows:

First, the center of the distribution curve in western China was not shifted significantly during the observation period. The height of its main peak mainly showed an evolutionary process of first rising and then falling. The width of its curve showed an evolutionary process of narrowing significantly and then widening slightly. The distribution of the right side of the curve increased slightly, which indicates that the overall trend of green economic efficiency of each city of western China has slightly improved. The absolute difference has a certain tendency to narrow.

Second, the distribution curve of green economic efficiency in western China had an obvious “right trailing” phenomenon. Its distribution extension showed a certain degree of contraction, which means that the gap between cities with high green economic efficiency and the mean for each city of western China is reduced.

Third, in terms of the wave evolution process, there is a persistent double peak in the green economic efficiency distribution in western China. The polarization characteristics have not been changed. In particular, one main single peak and one lower side peak mainly existed in the distribution of green economic efficiency in each city of western China in 2007, after which the peak value of the side peak of the curve decreased in 2013. Then, the peak gap between the main peak and the side peak of the curve narrowed in 2019, with a more obvious polarization trend. This indicates that a certain gradient of green economic efficiency in each city of western China emerged during the observation period, showing a weaker polarization trend.

As shown in Fig 5B and 5C, the center of the distribution curve in northwest China had no obvious displacement, while the center of the distribution curve in southwest China was slightly shifted to the right. The main peaks of the curves in both northwest China and southwest China showed a trend of first rising and then falling. The absolute differences of green economic efficiency in northwest China showed a slightly narrowing trend, while the absolute differences in southwest China showed a slightly widening trend. The right trailing phenomenon existed in both northwest China and southwest China. The green economic efficiency in northwest China showed a weak polarization trend, while southwest China showed a significant polarization.

### 5.3. Dynamic evolution of green social efficiency in western China

**Fig 6:**
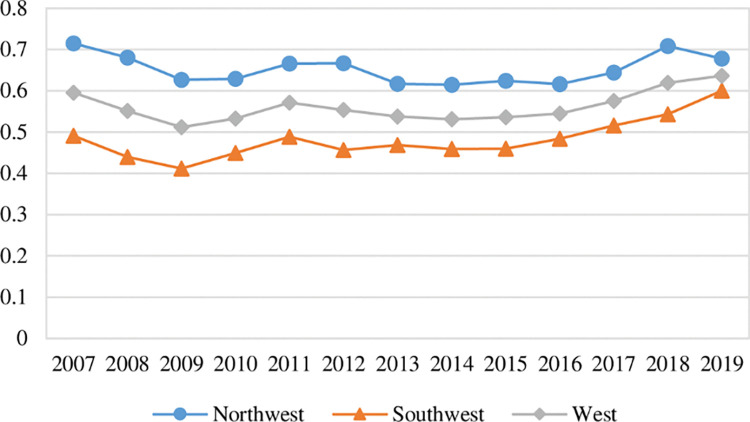
Trend of green social efficiency in western China.

Fig 6 shows the time-series trend of green social efficiency in each region of western China. It can be seen that:

First, in terms of the overall mean change, the mean value of green social efficiency in western China was 0.5952 in 2007, which declined to 0.5114 in 2009, and rose to 0.6361 in 2019, indicating that the mean of green social efficiency in western China showed an overall trend of first decreasing and then increasing and the change was not significant. During the observation period, the mean of green social efficiency in western China showed a fluctuating trend and an evolution process of first decreasing and then increasing. The same trend existed in southwest China and northwest China. However, in the later part of the observation period, the green social efficiency in northwest China increased rapidly and was higher than its mean in 2007, while the green social efficiency in southwest China increased more slowly and was lower than its mean in 2007.

Second, in terms of regional distribution, during the observation period, the mean of green social efficiency in southwest China was in the range of (0.4118, 0.5997). The mean of green social efficiency in northwest China was in the range of (0.6170, 0.7152). The mean of green social efficiency in western China was in the range of (0.5114, 0.6361). The green social efficiency of northwest China was higher than the mean of western China. The green social efficiency of southwest China was lower than the mean of western China. In 2019, the overall mean of green social efficiency in southwest China was 0.6781, which is higher than the overall mean of 0.5997 in northwest China. Overall, the gap between the green social efficiency of southwest China and northwest China was gradually narrowed.

**Fig 7:**
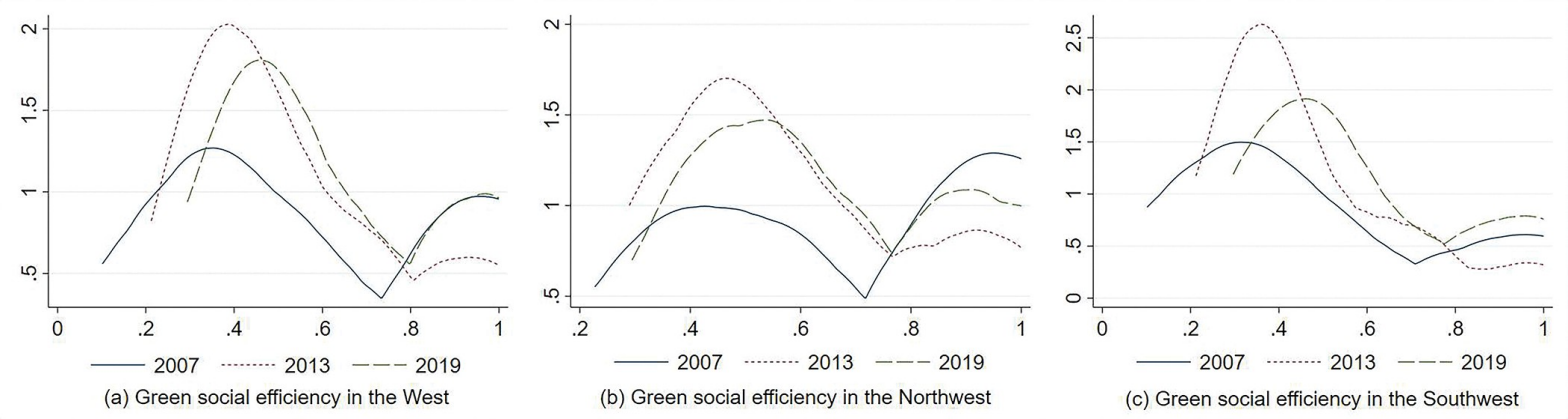
Kernel density curve of green social efficiency in western China.

Fig 7 illustrates the kernel density curves of green social efficiency in different regions of western China in different years. The specific distribution dynamics are characterized as follows:

First, the center of the distribution curve and the interval of change in western China gradually moved to the right during the observation period. Its main peak mainly showed the evolution process of first rising and then falling. The width of its curve showed a trend of gradual narrowing, which indicates that the overall green social efficiency of western China showed an obvious upward trend. Its absolute difference has a certain magnitude of narrowing trend.

Second, the distribution curve of green social efficiency in western China had an obvious “right trailing” phenomenon. Its distribution extension showed a certain degree of contraction, which means that the gap between cities with high green social efficiency and the mean of each city of Western China was reduced.

Third, in terms of the wave evolution process, the green social efficiency distribution in western China continued to have double peaks. The polarization characteristics tended to weaken. In which, the green social efficiency distribution in western China mainly shows a small difference between the main peak and the side peak in 2007. The polarization trend was more obvious. Then, the side peak of the curve decreased until 2013. The difference between the main peak and the side peak of the curve increased by 2019. The polarization trend tended to weaken, which indicated that the green social efficiency in western China showed a certain gradient effect and an obvious polarization trend during the observation period.

From Fig 7B and 7C, it can be seen that the centers of the distribution curves in both northwest China and southwest China moved slightly to the right. The main peaks of the ground curves in both northwest China and southwest China showed a rising and then falling trend. The absolute difference in green social efficiency in northwest China tended to decrease slightly, while the absolute difference in southwest China tended to increase slightly. The right trailing phenomenon existed in both northwest China and southwest China. The green social efficiency in northwest China showed an obvious polarization trend, while southwest China showed a weaker polarization phenomenon.

## 6. Discussion

### 6.1. Influencing factors

From the above, it can be seen that the three dimensions of green development efficiency in western China show a slight upward trend in general, but in terms of the efficiency values, only the green social efficiency is at a higher level in general, while the green ecological efficiency and the green economic efficiency are at a lower level in general. There is a tendency for the absolute difference of the three sub-efficiencies of the green development to be slightly reduced. Further, the results of green economic efficiency in western China is in line with the conclusions of the studies by Zhao (2020) and Liu (2020) [23,61]. The results of green social efficiency in western China are consistent with the conclusions of the study by Liang et al. (2023) [48]. Western China has invested a large amount of resources to protect local ecology, but due to the slow effect of ecological protection, its ecological protection effectiveness has only a small increase, especially in the northwest China [62,63], which is in line with the conclusions of green ecological efficiency in this study. Although the three sub-efficiencies of green development in this study cannot be compared with the single green development efficiency of the previous studies, the government can identify possible deficiencies in the local capacity for green development in ecological, economic and social terms from the differences in the results of the three sub-efficiencies of green development. Overall, there is much room for improvement in all three sub-efficiencies of green development in western China. It can be seen that all three sub-efficiencies of green development in western China have greater room for improvement. Then, what factors can promote or inhibit the green development efficiency in western China? Based on the existing literature, the level of economic development, the degree of government intervention, foreign direct investment, the level of financial development, population density, green technology innovation and other factors are several important factors affecting green development efficiency [42–46]. In this paper, the level of economic development (*lnpgdp*) is expressed in terms of the logarithm of real GDP per capita. Foreign direct investment (*lnfdi*) is expressed in terms of the logarithm of the amount of foreign direct investment actually utilized by each city. The degree of government intervention (*gov*) is expressed in terms of the ratio of local fiscal expenditures to GDP. The level of financial development (*fina*) is expressed in terms of the balance of loans from financial institutions in the end of the year on the proportion of GDP. Population density (*popu*) is the population density of the built-up area. Green technological innovation (*gtech*) is expressed by the number of green invention patents authorized in the city in that year. The data of prefecture-level cities in western China are obtained from China National Intellectual Property Administration, the China City Statistical Yearbook and China Urban and Rural Construction Statistical Yearbook [56,57,64].

Since the green ecological efficiency and green economic efficiency in western China lie between 0 and 2, and the green social efficiency lies between 0 and 1, it indicates that there is an obvious truncation of green development efficiency in western China, which cannot be accurately estimated by general econometric models. Therefore, this paper introduces the panel Tobit econometric model to estimate and analyze the influencing factors of green development efficiency in western China, and the model is set as follows:

$${GDE}_{it}=\beta_{0}+\beta_{1}{lnpgdp}_{it}+\beta_{2}{lnfdi}_{it}+\beta_{3}{gov}_{it}+\beta_{4}{fina}_{it}+\beta_{5}{popu}_{it}+\beta_{6}{gtech}_{it}+\varepsilon_{it}$$ (7)

Where *GDE*_*it*_ is the green development efficiency of each prefecture-level city in western China; *β*_0_ is the coefficient of the constant term; *β*_*i*_ is the regression coefficient of each influencing factor; *ε*_*it*_ is the random error term. In this paper, the model (7) is regressed with green ecological efficiency, green economic efficiency and green social efficiency as explanatory variables respectively. In this paper, the data of all the variables in the model are shrunk by 1%. Through the diagnosis of multicollinearity, the VIF of each explanatory variable is less than 2, indicating that there is no problem of multicollinearity among the influencing factors.

From the regression results in Table 6:

**Table 6:** Regression results of factors affecting green development efficiency in West China.

variable	Model (1) green ecological efficiency	Model (2) green economic efficiency	Model (3) green social efficiency
popu	0.0293** (0.0130)	-0.1068*** (0.0339)	0.0656*** (0.0339)
gov	-0.0714* (0.0393)	-0.3746*** (0.1018)	-0.5145*** (0.0903)
fina	0.0244*** (0.0085)	-0.0626*** (0.0211)	0.0806*** (0.0203)
lnpgdp	-0.0361*** (0.0065)	0.0441*** (0.0164)	0.0279*** (0.0154)
lnfdi	-0.0080*** (0.0016)	-0.0106** (0.0044)	-0.0040** (0.0044)
gtech	0.00004 (0.00004)	0.0008*** (0.0001)	0.0005*** (0.0001)
LR test P-value	0	0	0

Note

***, **, and * indicate passing 99%, 95%, and 90% significance level tests, respectively.

1. Population density (*popu*). In models (1) and (3), the regression coefficients of population density are significantly positive at the 5% and 1% levels, respectively, indicating that increasing the population density of the built-up area can significantly improve the green ecological efficiency and green social efficiency in western China. In model (2), the regression coefficient of population density is significantly negative at the 1% level, indicating that increasing the population density of the built-up area does not significantly improve the green economic efficiency in western China. This is consistent with the findings of Zhao et al. (2020) [23]. As an ecologically sensitive region, the concentration of the rural population in cities in western China, especially in the arid and semi-arid regions, can reduce the negative impact of the rural population on the ecological environment and the pressure on the utilization of ecological land. At the same time, it facilitates the centralization of the provision of green public services in the cities as well as the improvement of the quality of life. However, most cities in western China are slow in the green economic transformation. The increase in population density not only leads to a rapid rise in energy consumption, but also reduces the cost of labor in the cities, which is not conducive to the elimination of local outdated industries, and thus restricts the improvement of the green economy efficiency.
2. Degree of government intervention (*gov*). The regression coefficients of the degree of government intervention are significantly negative at the 10% and 1% levels, respectively, indicating that reducing the degree of government intervention in the market can significantly improve the green ecological efficiency, green economic efficiency and green social efficiency in western China. This is consistent with the findings of Yang et al. (2022) [65]. Excessive government intervention in the market will disrupt the market order, fail to regulate a series of problems brought about by market failures, and lead to ineffective allocation of resources, thereby inhibiting green development efficiency. Due to the impact of historical legacy issues, geographical conditions, development foundations, etc., the socio-economic development in western China, especially in remote and underdeveloped areas, is still mainly dependent on the local government to take the lead.
3. Financial development level (*fina*). In models (1) and (3), the regression coefficients of the level of financial development are significantly positive at the 1% level, indicating that improving the level of financial development can enhance the green ecological efficiency and green social efficiency in western China. In model (2), the regression coefficient of financial development level is significantly negative at the 1% level, indicating that increasing the level of financial development does not significantly enhance the green economic efficiency in western China. This is consistent with the findings of Cui and Wang (2023) [66]. As China’s credit funds are more favorable to large nationalized enterprises, it is easier for ecological construction projects, green public services and green municipal facilities construction projects to receive financial support from financial institutions, which in turn enhances green ecological efficiency and green social efficiency. Small and medium-sized enterprises, which are an important part of economic development in western China, face financing difficulties, which is not conducive to the active participation of small and medium-sized enterprises in the development of green products and the enhancement of green production capacity.
4. Economic development level (*lnpgdp*). In model (1), the regression coefficient of the level of economic development is significantly negative at the 1% level, indicating that increasing the level of economic development does not enhance the green ecological efficiency in western China. In models (2) and (3), the regression coefficients of the level of economic development are significantly positive at the 1% level, indicating that increasing the level of economic development can significantly enhance the green economic efficiency and green social efficiency in western China. This is consistent with the findings of Wang et al. (2022) [27]. Environmental governance, green facility construction, and efficiency improvement need to be supported by the level of economic development. Urban construction in western China attaches great importance to green development. The improvement of the level of economic development has a positive impact on the improvement of green economic efficiency and green social efficiency in western China. However, the extensive enterprises in western China still have resource waste and ecological damage, which are not conducive to the improvement of green ecological efficiency [4].
5. Foreign direct investment (*lnfdi*). The regression coefficients of FDI are significantly negative at the 5% and 1% levels, indicating that an increase in FDI reduces the green ecological efficiency, green economic efficiency and green social efficiency in western China, and that there is a “pollution paradise” in western China. This is consistent with the findings of Wang et al. (2022) and Wang et al. (2023) [27,67]. Due to the relatively low level of economic development in western China, local government has lowered the environmental standards in order to attract foreign investment, making the local area a “pollution haven” for multinational enterprises. The introduction of foreign investment has inhibited the green development efficiency in western China.
6. Green technology innovation (*gtech*). The regression coefficient of green technological innovation is significantly positive at the 1% level, indicating that encouraging green technological innovation can significantly enhance green economic efficiency and green social efficiency in western China. However, the effect of green technology innovation on the current green ecological efficiency in western China is not yet significant. This is consistent with the findings of Shang et al. (2022) [44]. Green technological innovation can promote new energy development, clean production, green product development and even green industry cultivation, and promote enterprises to optimize resource allocation, thus improving green economic efficiency. At the same time, green technological innovation can improve the ability of green public service capacity, urban domestic pollution control and recycling capacity, which thus improve green social efficiency.

### 6.2. Limitations and future prospects

This study also has certain limitations. Due to the immaturity of the theoretical framework of green development efficiency and the limitations of statistical data, the green eco-efficiency indicator system has not yet incorporated financial inputs for eco-protection, and the green social efficiency indicator system has not yet incorporated medical financial expenditure. Based on the ecological-economic-social system, it is still a great challenge to establish the indicator systems of green ecological efficiency, green economic efficiency and green social efficiency respectively to reflect the regional green development efficiency more comprehensively. The following aspects will be strengthened in the future: first, continue to carry out theoretical research on green ecological efficiency and green social efficiency, and further improve the indicator systems of these two types of efficiency; second, strengthen the research on the spatial differentiation characteristics of the three types of green development efficiency, and deeply analyze the regional differences, spatial linkages and the evolution of spatial characteristics.

## 7. Conclusions and recommendations

### 7.1. Conclusions

Based on the ecological-economic-social system, according to the theoretical framework of green development efficiency, the green development efficiency indicator system is constructed. The Super-SBM model, Super-SBM-Undesirable model containing undesired outputs, and kernel density estimation are used to measure the green development efficiency of 84 prefecture-level cities (including municipality) in western China from 2007 to 2019 and analyze its dynamic evolution. The following conclusions were obtained:

Firstly, green ecological efficiency and green economic efficiency in western China were generally at a low level, and mainly dragged by northwest China, while green social efficiency in western China was generally at a high level, and mainly dragged by southwest China.

Secondly, green ecological efficiency, green economic efficiency and green social efficiency in western China all show a trend of first slightly decreasing and then increasing. The trends in southwest China and northwest China are basically similar to those in western China.

Thirdly, the three sub-efficiencies of green development in western China all have a trend of small reduction in absolute difference, and all have right trailing and polarization phenomena. Particularly, their green social efficiency has a significant polarization phenomenon.

Fourthly, the lower green ecological efficiency in western China is due to the negative impacts from the level of government intervention, the level of economic development, and foreign direct investment. The lower green economic efficiency is due to the positive impacts from population density, the level of government intervention, the level of financial development, and foreign direct investment. The higher green social efficiency is due to the positive impacts from population density, the level of financial development, the level of economic development, and the green technological innovation.

### 7.2. Recommendations

Based on the above findings, the future improvement of green development efficiency in western China should be based on stimulating the endogenous power of green development, especially accelerating the improvement of green ecological efficiency and green economic efficiency in northwest China. In this paper, the following recommendations are made:

First, the development capacity of the green society in southwest China should be further enhanced. The government can gradually reduce its intervention in the market, utilize green finance, establish a social capital incentive mechanism for the key areas of green social development, encourage enterprises to participate in technological innovation in environmental governance, green infrastructure, and green public services, strictly limit investment in foreign high-pollution projects, improve the development of green industries, accelerate the construction of green infrastructure, speed up the construction of a green and low-carbon public service system, and actively create green office units and green communities.

Second, the government can improve the green development capacity of both ecological and economic aspects in northwest China. In terms of green ecology, the government can increase green financial support programs, accelerate key ecological protection projects, accelerate the construction of nature reserve systems and ecological carbon sink systems to improve the quality of the ecosystem as a whole, explore ways to realize ecological values, improve the linkage and collaboration mechanism for regional ecological protection, and improve the ecological protection input and evaluation system. In the green economy, the government can vigorously develop renewable energy, accelerate the cultivation of new industries such as cloud computing and high-end energy storage materials, and make good use of science and technology finance to support the development of science and technology enterprises, further give full play to the advantages of the natural environment and promote the development of regional specialty industries such as specialty animal husbandry, tourism, specialty plantation and specialty food processing industry, and raise the level of green economic development, use green finance to support enterprises’ participation in green technological innovation, speed up the introduction and research of green and low-carbon technologies, and accelerate the green and low-carbon transformation of production equipment and processes.

## Supporting information

S1 Data (XLSX)
